# Anxiety in adults with asthma during the coronavirus disease 2019 pandemic: a Canadian perspective

**DOI:** 10.1186/s13223-023-00833-z

**Published:** 2023-08-23

**Authors:** Sophia Linton, Kayley Xu, Lubnaa Hossenbaccus, Hannah Botting, Sarah Garvey, Adam Sunavsky, Lisa M. Steacy, Dean A. Tripp, Anne K. Ellis

**Affiliations:** 1 Department of Medicine, Queen’s University, Kingston, ON Canada; 2 Kingston General Health Research Institute-Allergy Research Unit, Kingston, ON Canada; 3 Kingston Health Science Centre-KGH Site, Kingston, ON Canada; 4 Department of Medicine, University of British Columbia, Vancouver, BC Canada; 5 Department of Psychology, Queen’s University, Kingston, ON Canada; 6 Department of Biomedical and Molecular Sciences, Queen’s University, Kingston, ON Canada

**Keywords:** Asthma, Anxiety, Allergies, COVID-19

## Abstract

**Background:**

Asthma is a chronic airway inflammatory disease that affects millions of Canadians and often contributes to higher levels of anxiety among patients. Since the coronavirus disease 2019 (COVID-19) pandemic was a time of increased anxiety and fear among the Canadian population, it was thought that those with asthma may experience heightened anxiety levels due to uncertain access to care, the potential to misinterpret asthma symptoms for symptoms of COVID-19 (or vice versa), and the concern about being treated differently by those around them when experiencing asthma symptoms. Therefore, this study sought to perform a cross-sectional analysis of the asthma-anxiety relationship in adults with and without asthma in the unique context of the COVID-19 pandemic from a Canadian perspective.

**Methods:**

This study employed the COVID-19 Associated Anxiety in Allergic Rhinitis and Asthma patients Experiencing Symptoms (CAAARES) survey, consisting of COVID-19-specific questions, the Generalized Anxiety Disorder Assessment-7 (GAD-7) and the Asthma Control Questionnaire-6 (ACQ-6). Data collection occurred through the Qualtrics XM platform and data analyses were conducted with the IBM SPSS Statistics 28 software.

**Results:**

A total of 741 valid responses were collected (asthma group, n = 244; control group, n = 497). 31.6% and 26.2% of respondents in the asthma and control groups, respectively, met the diagnostic criteria for GAD. There was no significant difference (p = .067) in mean GAD-7 scores between the two groups. A Hierarchal Multiple Regression (HMR) model was developed, and neither asthma status nor ACQ-6 score had a significant predictive effect on the GAD-7 score. There was a statistically significant (p < .001) weak positive correlation (r = .22) between GAD-7 and ACQ-6 scores. In a simple mediation (SMM) model, perceived COVID-19 stress of others was not identified as a significant mediator of the relationship between ACQ-6 and GAD-7 (indirect effect β = 0.014).

**Conclusion:**

Our study of a Canadian cohort demonstrates elevated levels of anxiety overall, amongst both asthma and control groups. While AR status was significantly greater in the asthma group, it was not a significant predictive variable of GAD-7 score. Our data suggests that COVID-19-specific factors appear to have a greater contribution to anxiety than asthma status or control.

## Background

Asthma is estimated to affect 3.8 million Canadians, making it the third most common chronic disease nationally [1]. The Global Initiative for Asthma (GINA) defines asthma as a “heterogenous disease, usually characterized by chronic airway inflammation. It is defined by a history of respiratory symptoms, such as wheeze, shortness of breath, chest tightness and cough, that vary over time, together with variable expiratory airflow limitation.”[2] Well known triggers of asthma include exercise, allergen or irritant exposure, weather, or viral respiratory infections. Allergic rhinitis (AR) or “hay fever” is a common comorbidity of asthma that occurs in over 80% of patients with asthma and can increase asthma severity [3].

Studies indicate that there is no increased risk of death caused by Coronavirus disease 2019 (COVID-19) following infection of the SARS-COV-2 virus, in people with mild to moderate, well-controlled asthma [4–6]. However, people using oral corticosteroids to treat their asthma[4, 7] or who are hospitalized with severe asthma[8] have been shown to be at a greater risk of COVID-19 related death. Further challenges exist in the identification and treatment of COVID-19 and asthma due to their overlapping symptom profiles [9]. Several joint statements have also been published regarding the impact of COVID-19 on asthma and advice on asthma management [2, 10].

There is a two-way relationship between asthma and anxiety and depression that is well described. Studies show that individuals with asthma are more likely to develop anxiety and depressive disorders[11–13] and that the presence of these disorders negatively impacts asthma control, lung function, treatment response, and quality of life [14, 15]. Additionally, emotional stress and panic attacks can lead to asthma exacerbations in children and adults and negatively impact medical adherence [16–18]. GINA recommends arranging a mental health assessment for patients exhibiting symptoms of anxiety or depression [2]. The impact of comorbid AR on asthma-related anxiety is unknown. Sleep disturbances and sleep apnoea due to obstructed airways are common in this population, which can negatively affect quality of life and lead to depression [19–21].

The COVID-19 pandemic has undoubtedly impacted the mental health of the general population [22]. Increased levels of anxiety, depression, and insomnia are expected, especially in people with pre-existing medical conditions that increase their risk of COVID-19 hospitalization or death, such as moderate-to-severe or uncontrolled asthma [8, 23]. Other COVID-19 specific concerns may increase pandemic-time anxiety for people with asthma, including limited access to care, the potential to misinterpret asthma symptoms for symptoms of COVID-19 (or vice versa), and the concern about being treated differently by those around them when experiencing asthma symptoms. These factors, compounded with a potential underlying asthma-anxiety relationship, make understanding the asthma-anxiety relationship within the context of COVID-19 a priority.

There are some current reports of increased COVID-19 related anxiety in adults with asthma. Wei et al. collected 10,760 responses to the COVID-19 Household Impact Survey Data from April-June 2020 and found that adults with asthma were more likely to report feeling nervous, anxious or on edge, depressed, hopeless about the future, and to have a physical reaction when thinking about their experiences during the COVID-19 pandemic in the past 7 days compared to adults without asthma [24]. The associations of COVID-19 anxiety with asthma control in adults were evaluated by Eldeirawi et al. using the Coronavirus Health and Impact Survey Initiative and the Asthma Control Test (ACT). Participants with high anxiety were twice as likely to have uncontrolled asthma compared with counterparts reporting low levels of anxiety [25]. One group also reported on the relationship between asthma treatment with biologics and anxiety levels during the pandemic, specifically psychological distress, anxiety, depression, and suicidal risk in severe asthma patients. The study reported a significant improvement in all observed parameters, including ACT, stress, anxiety, and depressive symptoms despite the COVID-19 pandemic [26].

To our knowledge, there are no studies investigating the impact of COVID-19 on the asthma-anxiety relationship in a Canadian population compared to a control or non-asthmatic group. Further, there are no studies that take into consideration AR status. Therefore, in this study, we aim to assess the impact of the COVID-19 pandemic on anxiety levels in adults with and without asthma, hypothesizing that anxiety levels would be more elevated in adults with asthma than those without.

## Methods

### Survey design

The COVID-19 Associated Anxiety in Allergic Rhinitis and Asthma patients Experiencing Symptoms (CAAARES) survey, conducted online through the ‘Qualtrics XM’ platform, was used for data collection. The CAAARES survey was distributed to participants sourced from a Kingston Allergy Research database in addition to a broader population, mainly focused on Southeastern Ontario, through promotion on several social media outlets. There was no prior knowledge of the prevalence of anxiety in the participant database at the time that the survey was distributed. Responses were collected between April to August 2020, which captured the spring and summer allergy season, when weed pollen, tree pollen, and mold spores are at their annual peak levels overlapping with the initial waves of the COVID-19 pandemic. All participants gave their informed consent by reviewing a Letter of Information and agreeing to participate in the study. The study was reviewed for ethical compliance by the Queen’s University Health Sciences and Affiliated Teaching Hospitals Research Ethics Board.

### Participants

**Fig. 1:**
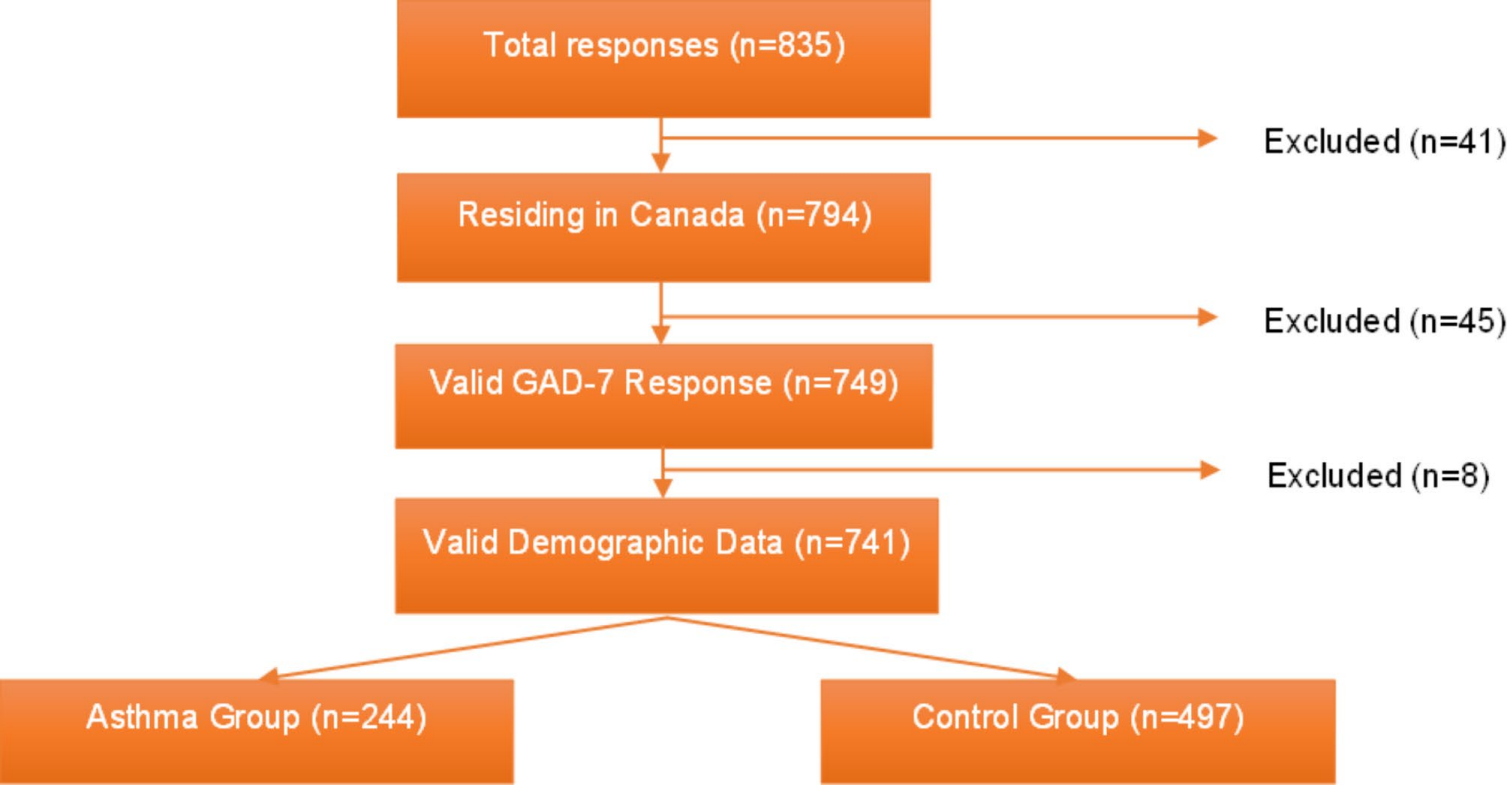
Flow Chart of Survey Responses. Flow chart of included and excluded responses from the first iteration of the CAAARES survey forming the present study population. CAAARES, COVID-19 Associated Anxiety in patients with Asthma and AR Experiencing Symptoms; COVID-19, coronavirus disease 2019; AR, allergic rhinitis; GAD-7, Generalized Anxiety Disorder Assessment-7

835 adult participants completed the first iteration of the CAAARES survey. Participants were excluded if they failed to report a location of residence within Canada or if they failed to report a valid response to GAD-7, demographic data (gender, age, employment status, AR status, previous anxiety diagnosis, history of access to mental health services), or asthma diagnosis (Fig. 1).

### Measures

Anxiety was measured using the Generalized Anxiety Disorder Assessment-7 (GAD-7), a self-reported questionnaire to screen for Generalized Anxiety Disorder (GAD) and assess severity [27]. Participants rated the severity of anxiety symptoms over the previous 2 weeks on a scale of 0 (*Not at all sure*) to 3 (*Nearly everyday*). Scores were calculated by summing items, with higher GAD-7 scores indicating greater severity. A score of 10 or higher is suggested as the diagnostic threshold for GAD [27].

Asthma control was measured with the Asthma Control Questionnaire-6 (ACQ-6), a self-reported questionnaire assessing participant’s asthma control in the preceding week [28, 29]. Participants rated six sections (nighttime awakenings, symptoms upon awaking in the morning, impact on activity, shortness of breath, wheezing, and short acting bronchodilator use). Scores were calculated by determining the mean of all items, with greater scores indicating poorer asthma control.

### Data analysis

Participant responses with missing input for variables being tested were selectively excluded to maximize sample size and power of statistical tests being conducted. All analyses, including Chi square tests, *t* tests, regressions, and correlations, used a *p* value cut-off of *p* ≤ .05 in determining statistical significance. All regression coefficients reported are standardized. All participant data were deidentified. All analyses were conducted with IBM SPSS Statistics 28.

## Results

### Cross-sectional analysis

A cross-sectional analysis was conducted to compare anxiety in a group of participants with asthma with that of a control group, defined as participants with or without AR self-identifying as not having asthma. A total of 741 responses were included, 244 participants with asthma (32.9% of total included responses) and 497 participants without asthma (67.1% of total included responses). The baseline demographic characteristics of the asthma group and the control group are summarized in Table 1. Chi square testing showed that more participants in the asthma group had AR and previous anxiety syndrome diagnosis compared with the control group.

**Table 1:** Baseline demographic characteristics of the asthma group and the control group including comparisons with Chi square tests. Values in red indicate results showing a statistically significant difference between groups

	n (% of group)		
***Characteristic***	**Asthma Group (n = 244)**	**Control Group (n = 497)**	**Chi Square Results**
***Gender***			*p = .281*
*Man*	37 (15.2)	97 (19.5)	
*Woman*	203 (83.2)	395 (79.5)	
*Non-binary/prefer not to disclose*	4 (1.6)	5 (1.0)	
***Age***			*p = .480*
*18–25*	36 (14.8)	97 (19.5)	
*26–35*	61 (25.0)	116 (23.3)	
*36–45*	58 (23.8)	117 (23.5)	
*46–55*	43 (17.6)	94 (18.9)	
*56–65*	39 (16.0)	59 (11.9)	
*≥ 65*	7 (2.9)	14 (2.8)	
***Employment Status***			*p = .186*
*Full time*	64 (26.2)	137 (27.6)	
*Full time working from home*	38 (15.6)	102 (20.5)	
*Part time*	12 (4.9)	35 (7.0)	
*Part time working from home*	10 (4.1)	21 (4.2)	
*Unemployed*	8 (3.3)	21 (4.2)	
*Recently laid off due to COVID*	37 (15.2)	47 (9.5)	
*Retired*	23 (9.4)	35 (7.0)	
*Student*	22 (9.0)	52 (10.5)	
*Other*	30 (12.3)	47 (9.5)	
***Allergic Rhinitis Status***			*p < .001*
*Yes*	222 (91.0)	377 (75.9)	
*No*	22 (9.0)	120 (24.1)	
***Previous Anxiety Syndrome Diagnosis***			*p = .034*
*Yes*	141 (57.8)	246 (49.5)	
*No*	103 (42.2)	251 (50.5)	
***Currently Receiving Mental Health Services***			*p = .805*
*Yes*	31 (12.7)	60 (12.1)	
*No*	213 (87.3)	437 (87.9)	
***Received Mental Health Services in the Past***			*p = .424*
*Yes*	145 (59.4)	280 (56.3)	
*No*	99 (40.6)	217 (43.7)	

**Fig. 2:**
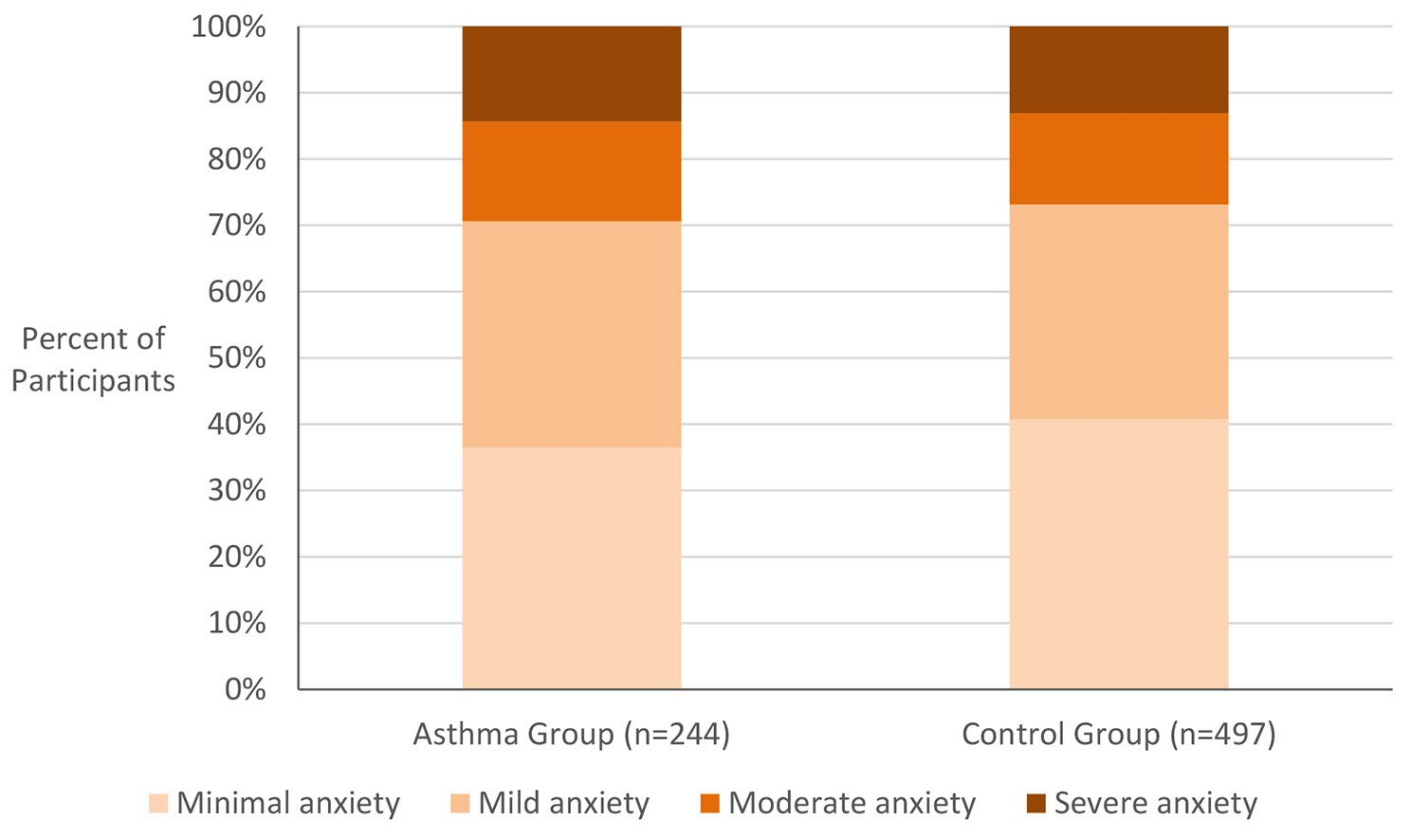
Anxiety Levels Among Participants With Asthma and Without Asthma. High levels of anxiety were found in both the asthma group (n = 244) and the control group (n = 497). Red boxes indicate Generalized Anxiety Disorder Assessment-7 (GAD-7) scores meeting diagnostic criteria for generalized anxiety disorder (GAD-7 ≥ 10). There was no significant difference in mean GAD-7 scores between groups (t(739) = 1.83, p = .067)

Figure 2 compares the percentage of participants in the asthma and control groups reporting GAD-7 scores indicating minimal anxiety (GAD-7 0–4), mild anxiety [5–9], moderate anxiety [10–14], and severe anxiety [15–21]. Participants meeting the diagnostic criteria for GAD are indicated by the red boxes (GAD-7 score ≥ 10). In the asthma group, 31.6% of respondents met the diagnostic criteria for GAD, compared with 26.2% in the control group. The mean GAD-7 scores in the asthma and control groups were 7.50 (SD = 5.80) and 6.70 (SD = 5.47), respectively. There was no significant difference in mean GAD-7 scores between the two groups when compared with a two-tailed t-test (t(739) = 1.83, p = .067).

To assess the impact of asthma status on GAD-7 scores while controlling for other variables, a hierarchal multiple regression (HMR) was conducted, as summarized in Table 2. Predictor variables (demographic variables and COVID-19-specific parameters) were included in the model if they were determined to have a significant predictive effect on GAD-7 score in bivariate analyses or if they were not balanced between the asthma and control groups at baseline as determined by Chi square testing (Table 1). Predictor variables meeting this inclusion criteria included sex, age, asthma status, AR status, previous anxiety syndrome or symptom diagnosis, current access of mental health services (MHS), past access of MHS, general COVID-19-related worry, and perceived COVID-19 stress of others. Sex was coded as a binary variable and participants who did not identify with the gender binary or who preferred not to disclose their gender identity were not included owing to insufficient sample size (Table 1). The parameter of general COVID-19-related worry was assessed by asking respondents how they felt about the current situation with COVID-19 in general (not worried, somewhat worried, worried, or extremely worried). To assess the parameter of perceived COVID-19 stress of others, respondents were asked to separately rate the level of stress associated with COVID-19 they believed others around them were experiencing (family, relatives they did not live with, and close friends) on a Likert scale from 0 to 10 (10 being the most worried). The mean of these 3 values formed the composite measure of perceived COVID-19 stress of others, which had good internal consistency when tested by Cronbach’s alpha (α = 0.79). After filtering for survey responses with valid responses to the included parameters, the sample size of the HMR model was n = 704.

**Table 2:** Results of the Hierarchal Multiple Regression (HMR) with Generalized Anxiety Disorder Assessment-7 (GAD-7) score as the independent variable (n = 704). Asthma status did not add to the predictive value of Model 1a when included in Model 1b (ΔR^2^ = 0.00, F(1,694) = 0.023, *p = .879*)

Predictor Variables	Model 1a				Model 1b			
		**95% Confidence Intervals**				**95% Confidence Intervals**		
	β	**Lower Bound**	**Upper Bound**	***p*** **Value**	β	**Lower Bound**	**Upper Bound**	***p*** **Value**
Gender (0 = Man, 1 = Woman)	0.02	-0.60	1.13	0.543	0.02	-0.60	1.13	0.546
Age	-0.18	-0.96	-0.47	< 0.001	-0.18	-0.96	-0.47	< 0.001
Allergic Rhinitis status (0 = N, 1 = Y)	0.01	-0.67	1.04	0.666	0.01	-0.69	1.04	0.688
Previous anxiety diagnosis (0 = N, 1 = Y)	0.36	3.30	4.79	< 0.001	0.36	3.29	4.79	< 0.001
Current access of Mental Health Services (MHS) (0 = N, 1 = Y)	0.06	-0.04	2.08	0.059	0.06	-0.04	2.09	0.059
Past access of MHS (0 = N, 1 = Y)	0.05	-0.20	1.31	0.151	0.05	-0.20	1.31	0.150
General COVID-19-related worry	0.16	0.61	1.36	< 0.001	0.16	0.60	1.36	< 0.001
Perceived COVID-19 stress of others	0.24	0.49	0.85	< 0.001	0.24	0.49	0.85	< 0.001
Asthma status (0 = N, 1 = Y)	--	--	--	--	0.00	-0.66	0.77	0.879
*Predictor variables significant at p ≤ .05 are indicated in red.*								

Table 2 displays the results of this HMR model. The overall regression was statistically significant with R^2^ = 0.39 (F(8,695) = 55.26, *p < .001*), indicating that the model provided a reasonable fit for predicting GAD-7 scores. After asthma status was added as a predictor variable to Model 1a to construct Model 1b, there was no change in the predictive value of the model ΔR^2^ = 0.00, F(1,694) = 0.023, *p = .879*). In other words, asthma status did not have a significant predictive effect on GAD-7 score after controlling for the effect of covariates (β = 0.00, *p = .879*).

### Subgroup Analysis

A subgroup analysis of cases (n = 231) was conducted to explore factors associated with anxiety within the asthma group, including the asthma burden of disease and COVID-19-specific concerns. The mean ACQ-6 score was 1.02 (SD = 0.94). To assess the relationship between burden of disease and anxiety, the linear correlation between GAD-7 score and the ACQ-6 score was calculated. There was a statistically significant weak positive correlation between GAD-7 score and ACQ-6 score (r(244) = 0.22, p < .001).

To assess the impact of asthma burden of disease on anxiety while controlling for the effect of other variables, a second HMR was conducted within the asthma subgroup as summarized in Table 3. Predictor variables again were included if they were determined to have a significant predictive effect on GAD-7 scores within the asthma subgroup in bivariate analyses. These predictor variables were sex, age, previous anxiety syndrome or symptom diagnosis, current access of MHS, past access of MHS, general COVID-19-related worry, perceived COVID-19 stress of others, being treated differently by others, worry about the asthma-COVID-19 symptom overlap, and worry about increased COVID-19 risk. The parameter of being treated differently by others was assessed by a dichotomous yes or no question asking respondents if their friends or family treated them differently when they showed asthma symptoms. The parameter of worry about the asthma-COVID-19 symptom overlap was assessed by asking participants how worried they were that they would mistake their normal asthma symptoms for symptoms of COVID-19 (not worried, somewhat worried, worried, or extremely worried). Finally, the parameter of worry about increased COVID-19 risk was assessed by asking participants how worried they were that having asthma would increase their risk of developing serious symptoms from COVID-19 (not worried, somewhat worried, worried, or extremely worried). After the exclusion of survey responses without valid responses to the included parameters, the sample size of the subgroup HMR was n = 231.The overall regression was statistically significant with R2 = 0.39 (F(9,221) = 15.41, p < .001), indicating that the model provided a reasonable fit for predicting GAD-7 scores. After ACQ-6 score was added as a predictor variable to Model 2a to construct Model 2b, there was no change in the predictive value of the model ΔR2 = 0.007, F(1,220) = 2.48, p = .117). In other words, ACQ-6 score did not have a significant predictive effect on GAD-7 score after controlling for the effect of covariates within the asthma subgroup (β = 0.09, p = .117).

**Table 3:** Results of a Hierarchal Multiple Regression (HMR) model within the asthma subgroup, with Generalized Anxiety Disorder Assessment-7 (GAD-7) score as the independent variable (n = 231). Asthma Control Questionnaire-6 (ACQ-6) scores did not significantly improve the predictive value of Model 2a after being added in Model 2b (ΔR^2^ = 0.007, F(1,220) = 2.48, *p = .117*)

Predictor Variables	Model 2a				Model 2b			
		**95% Confidence Intervals**				**95% Confidence Intervals**		
	β	**Lower Bound**	**Upper Bound**	***p*** **Value**	β	**Lower Bound**	**Upper Bound**	***p*** **Value**
Gender (0 = Man, 1 = Woman)	0.03	-1.32	2.17	0.633	0.03	-1.34	2.15	0.649
Age	-0.16	-1.15	-0.2	0.005	-0.16	-1.17	-0.23	0.004
Previous anxiety diagnosis (0 = N, 1 = Y)	0.42	3.61	6.4	< 0.001	0.41	3.43	6.24	< 0.001
Current access of Mental Health Services (MHS) (0 = N, 1 = Y)	0.03	-1.42	2.57	0.570	0.03	-1.39	2.59	0.550
Past access of MHS (0 = N, 1 = Y)	0.04	-0.97	1.86	0.539	0.04	-0.96	1.86	0.527
General COVID-19-related worry	0.06	-0.39	1.11	0.340	0.07	-0.34	1.16	0.277
Perceived COVID-19 stress of others	0.23	0.36	1.03	< 0.001	0.24	0.37	1.04	< 0.001
Worry about symptom overlap	0.02	-0.6	0.85	0.740	-0.01	-0.84	0.69	0.851
Worry about increased COVID-19 risk	0.08	-0.24	1.16	0.198	0.07	-0.31	1.09	0.276
ACQ-6 Score	--	--	--	--	0.09	-0.14	1.3	0.117
*Predictor variables significant at p ≤ .05 are indicated in red.*								

**Fig. 3:**
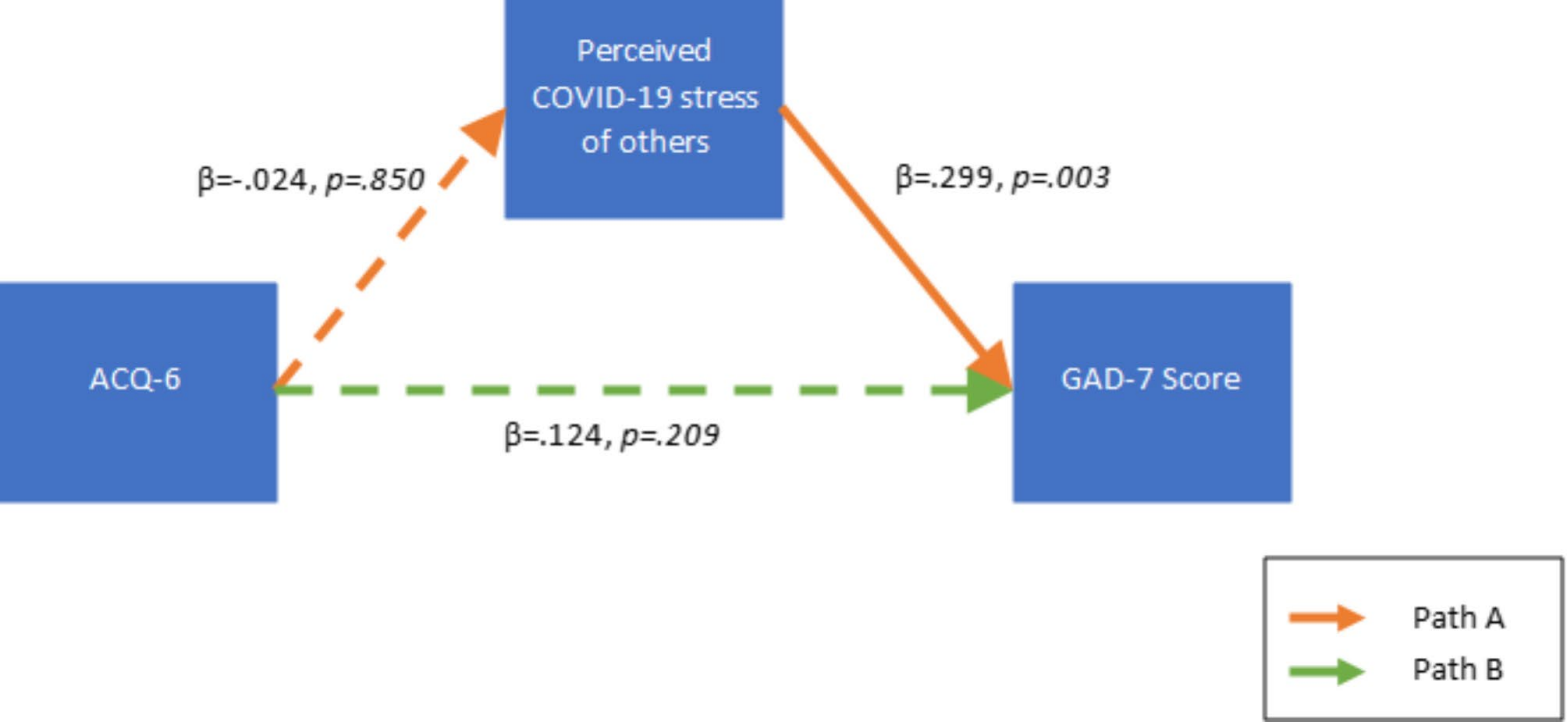
Impact of Asthma Control on GAD-7 and perceived COVID-19 stress of others. Simple mediation model with Asthma Control Questionnaire-6 (ACQ-6) score as the independent variable, Generalized Anxiety Disorder Assessment-7 (GAD-7) score as the dependent variable, and perceived COVID-19 stress of others as a mediator in the subgroup of participants with poorly controlled asthma (defined as ACQ-6 ≥ 1.5). Covariates included age and previous anxiety syndrome diagnosis. Solid lines indicate statistically significant effects and dashed lines indicate statistically insignificant effects. Perceived COVID-19 stress was not a significant mediator of the relationship between ACQ-6 and GAD-7 (indirect effect β=-0.007, 95% CI=[-0.081,0.085]).

The perceived COVID-19 stress of others was identified as a significant predictor of anxiety in asthma participants in Model 2b of the subgroup HMR. To further investigate the impact of this COVID-19-specific parameter on the relationship between ACQ-6 score and GAD-7 score, a simple mediation model was tested using Hayes’s PROCESS with 95% confidence intervals (CIs), 10,000 bootstrap samples, and standardized regression coefficients. Age and previous anxiety syndrome or symptom diagnosis were included as covariates in the mediation model because they had significant predictive effects in Model 2b. After the exclusion of survey responses without valid responses to the included parameters, the sample size of the subgroup mediation model was n = 242. With the model including ACQ-6 as the independent variable, GAD-7 score as the dependent variable, and the perceived COVID-19 stress of others as a mediator, it did not show that perceived COVID-19 stress was a significant mediator of the relationship between ACQ-6 and GAD-7 (indirect effect β = 0.014, 95% CI=[-0.020,0.048]). Finally, we sought to understand whether individuals presented ifferentlyin terms of GAD-7 or perceived COVID-19 stress of others depending on their asthma control (Fig. 3). We defined poor asthma control as an ACQ-6 score ≥ 1.5 (n = 67). Perceived COVID-19 stress was not a significant mediator of the relationship between ACQ-6 and GAD-7 (indirect effect β=-0.007, 95% CI=[-0.081,0.085]) with ACQ-6 as the independent variable, GAD-7 score as the dependent variable, and the perceived COVID-19 stress of others as a mediator.

## Discussion

In our study, we found similarly high levels of pandemic-time anxiety in adults with and without asthma in a Canadian population. A total of 31.6% of respondents in the asthma group met the diagnostic criteria for GAD, which is comparable to other estimates of 48% using the Coronavirus Health and Impact Survey Initiative[25] and much higher than pre-pandemic estimates of 13.7% using the Hospital Anxiety and Depression Scale [14]. A total of 26.2% of the control group met the diagnostic criteria for GAD, evidently higher than pre-pandemic estimates of 2.5% GAD prevalence among Canadians [30].

Most participants from both groups reported they had received mental health services in the past (56–60%, n = 425). Despite high levels of anxiety and depression reported, only 12–13% of all participants reported currently receiving mental health services at the time our survey was collected. This seemingly contradictory phenomenon is consistent with other reports. A systematic review by Yonemoto et al. investigated help-seeking behaviours for mental health problems during the COVID-19 pandemic and found that most studies reported delays, decrease, or deficits in help-seeking behaviours among a wide variety of individuals [31]. The reason for these changes is multifaceted (societal and familial stigma, lack of accessibility and/or affordability, negative attitudes/poor experiences, time constraints, etc.) but ultimately may have resulted in lost opportunities to link patients with appropriate treatment which reinforced high levels of anxiety and depression, as seen in our study [31].

In a Canadian context, our asthma group reported higher GAD-7 scores than the control group, with ~ 32% meeting diagnostic criteria for GAD. Yet, there was no significant difference between the two groups (p = .067), indicating this heightened anxiety was experienced by the general population. The high levels of anxiety seen in asthma group are close to those reported in the literature. The study by Eldeirawi et al. found that out of 873 survey responses from adults diagnosed with asthma, ~ 48% of them had a high anxiety score. Participants with higher anxiety levels were also more likely to report having uncontrolled asthma – 57% had a self-reported asthma attack during the pandemic, 29% contacted their healthcare provider for urgent symptoms and 43% had uncontrolled asthma [25]. Another study by Lacwik et al. performed a linear regression analysis showing that both state and trait anxiety were significantly associated with the change in ACQ (P < .001 and P < .01, respectively) [32]. Our study reported asthma control using the ACQ-6 score, which uses a 7-point scale where a score of 6 represents maximum impairment for symptoms and rescue use [28]. The mean score was 1.02 (SD = 0.94), indicating that overall, the asthma group had well-controlled asthma. Still, we found a statistically significant weak positive correlation between the GAD-7 score and the ACQ-6 score (p < .001), suggesting there is a relationship between anxiety level and asthma control.

To assess the impact of COVID-19 on anxiety in the total study population (n = 704) and the asthma group(n = 231), HMR models were developed with GAD-7 as the independent variable. Within the total study population, asthma status was not a statistically significant predictor variable of GAD-7 scores. Similarly, the ACQ-6 score did not have a significant predictive effect on GAD-7 scores after controlling for the effect of covariates within the asthma subgroup (β = 0.09, p = .117). However, age, previous anxiety diagnosis, and, most remarkably, perceived COVID-19 stress of others were statistically significant (p < .05) predictor variables in both models. In the HMR model, an additional COVID-19 factor, “general COVID-19-related worry”, was also a statistically significant predictor variable. From these results, we can conclude that within the context of the pandemic and our study population, COVID-19-specific factors appear to have a greater contribution to anxiety than asthma status or control. A similar effect was seen in a study by our group that investigated anxiety in adults with AR during the pandemic. Xu et al. reported that AR status had no significant predictive effect on GAD-7 in a HMR model (ΔR2 = 0.00, P = .69).

Several studies have confirmed that AR can negatively impact sleep and anxiety, among other psychiatric conditions [33–35]. In the current study, the asthma group had significantly higher rates of AR, but AR status did not serve as a significant predictor of anxiety in the HMR models. It is plausible to consider that the high pandemic-time anxiety experienced by the asthma group overshadowed the impact of comorbid AR on anxiety levels. Other studies have investigated pandemic-related anxiety in people with AR with conflicting findings. Ekström et al. explored anxiety and stress in relation to COVID-19 among young adults and the potential influence of asthma and AR. In their study, symptoms of AR were also not associated with increased concern or anxiety in relation to COVID-19 [36]. Meanwhile, Wang et al. conducted a survey study of 98 participants with AR and 56 healthy controls in Wuhan, China, finding that participants with AR reported more anxiety than healthy controls [37]. Future research into anxiety levels in individuals with comorbid AR and asthma would be of value to clarify this conflict in the literature.

The asthma-anxiety relationship was captured by the CAAARES questionnaire, specifically through the GAD-7 and ACQ-6 with COVID-19-related questions. However, the risk or presence of depression, insomnia, or other psychiatric conditions were not captured in our study. There is strong evidence to suggest the comorbidity of asthma and depression. A large pre-pandemic meta-analysis by Jiang et al. found that the prevalence of asthma in people with depression is much higher than it is in general population with the odds ratio of 3.17 (95% CI 2.82–3.56, p < .00001) and that people with asthma have significant higher risks of having depression than healthy controls (*OR* = 1.52; 95% CI, 1.30 to 1.79; *p* < .00001) [11]. A similar study from the USA captured 20,272 responses using the National Health Nutrition Examination Survey and also found that people with major depression had 3.4 times higher odds of asthma than did those with minimal or no depressive symptoms (95% CI, 2.6–4.5; P < .01) [15]. Among asthma and chronic obstructive pulmonary disorder patients in the context of the COVID-19 pandemic, a study by Pedrozo-Pupo and Campo-Arias found a 10.6% prevalence of high COVID-19 perceived stress, 11.3% post-traumatic stress risk, 31.5% depression risk, and 57.7% insomnia risk [38]. More research on depression and asthma in the COVID-19 era are desperately needed.

A major limitation to this study is that no data were collected about pre-pandemic levels of anxiety or asthma disease burden. It was therefore not possible to compare pandemic-time GAD-7 and ACQ-6 scores with a baseline. de Boer et al. compared fear, anxiety, and depression in people with asthma versus controls between pre-COVID-19 and during COVID-19 lockdown with a cross-sectional online survey. During the pandemic, patients with asthma displayed a clinically relevant increase in anxiety (3.32 ± 2.95 vs. 6.68 ± 3.78; p < .001) and depression (1.30 ± 1.15 vs. 3.65 ± 3.31; p < .001), according to the Hospital Anxiety and Depression Scale scores compared to pre-COVID-19 assessment, a finding that was not seen in the control group [39]. Other limitations to this study include the potential for measurement bias due to the lack of formal testing of questions assessing COVID-19-specific parameters and the lack of equal sample size between the asthma and control groups; however, this was simply the nature of the population of respondents.

## Conclusion

Our study of a Canadian cohort demonstrates elevated levels of anxiety among both asthma and control groups. While AR status was significantly greater in the asthma group, it was not a significant predictive variable in GAD-7 score. In the context of the pandemic and our study population of a Canadian cohort, COVID-19-specific factors appear to have a greater contribution to anxiety than asthma status or control. Physicians need to be aware of the potential development of anxiety in patients with asthma and other chronic respiratory conditions, especially as they pertain to pandemics.

## Data Availability

Data sharing is not applicable to this article as no datasets were generated or analysed during the current study.

## Abbreviations

COVID-19: coronavirus disease 2019
AR: allergic rhinitis
CAAARES: COVID-19 Associated Anxiety in Allergic Rhinitis and Asthma patients Experiencing Symptoms
GAD-7: Generalized Anxiety Disorder Assessment-7
ACQ-6: Asthma Control Questionnaire-6
HMR: Hierarchal Multiple Regression
SMM: simple mediation model
GINA: Global Initiative for Asthma
GAD: Generalized Anxiety Disorder
